# Incidence of Parkinson Disease in Wakayama, Japan

**DOI:** 10.2188/jea.12.403

**Published:** 2007-11-30

**Authors:** Seiji Morioka, Kiyomi Sakata, Sohei Yoshida, Ekini Nakai, Mitsuru Shiba, Noriko Yoshimura, Tsutomu Hashimoto

**Affiliations:** 1 Department of Public Health, Wakayama Medical University.; 2 Koza Public Health Centre.; 3 Department of Neurology, Wakayama Medical University.; 4 Department of Neurosurgery, Wakayama Medical University.; 5 Department of Psychiatry, Wakayama Medical University.

**Keywords:** Parkinson disease, incidence, Wakayama, mail survey

## Abstract

To estimate an accurate annual incidence of Parkinson disease in Wakayama, a mail survey was conducted in 1998. A questionnaire was delivered to each clinic where Parkinson disease would be potentially diagnosed. The survey was conducted in February 1998 by the Research Committee on Parkinson disease of Wakayama. A total of 792 clinics and 87 hospitals were listed as candidates. Physicians in these 879 medical facilities were asked and instructed to register all newly diagnosed patients with Parkinson disease in 1997 according to the diagnostic criteria proposed by the Japanese Research Committee on Neuro-degenerative Diseases. Of 879 facilities, 873 ones including 81 hospitals replied (response rate: 99%). A total of 229 patients were reported as newly diagnosed cases in 1997. Of these cases, 183 cases were classified as Yahr I to III. The annual incidence rate was 16.9 per 100,000 population (95% confidence interval: 14.5-19.3). Male-to-female ratio was 1:1.4 as a whole, and the dominant age stratum was 70 to 79 years old. When Parkinson disease incidence was observed from northern part of Wakayama to south by district, crude rates (95% CIs) were 15.9(12.9-18.9), 18.1(12.0-24.2), and 19.3(13.4-25.2). After age-adjustment using the Japanese Model Population in 1985, differences of Parkinson disease incidence became attenuated and adjusted rates (95% CIs) turned to 10.8(9.1-12.7), 10.4(8.6-12.2), and 9.9(6.9-12.9), respectively.

In Japan, etiological mechanism of subacute myelo-optico neuropathy was successfully elucidated in 1970 by an epidemiologic research committee which the Japanese Ministry of Health and Welfare of Japan sponsored. From then on, the Ministry has specified some 40 chronic diseases which were etiologically unknown, uncommon, incurable, and care-laden as ‘intractable diseases’ to promote research projects on those patients as well as to provide financial and medical supports.^1^ Parkinson disease (PD) was added to the intractable diseases in 1978. Since the prevalence of PD was thought to be relatively high, the Ministry restricted financial supports only to severe cases, namely grade III,IV, and V by Yahr classification.^2^ Therefore, no accurate information has been available on PD incidence based on financially certified PD cases in Japan.

Wakayama Prefecture, Japan had around 1.08 million population in 50 municipalities by the National Census in 1995, and the pace of ageing has been quite rapid. PD is a major target of public health administration, but no estimation has been made for PD incidence in Wakayama. Kasamatsu et al. found an increasing tendency in prevalence of financially certified PD cases in 1984 from north to south in Wakayama.^3^ However, the study provided only limited information since PD cases in the study were classified as Yahr III, IV, and V only. It is therefore essential to obtain a precise annual incidence of PD for both further research and administration.

Furthermore, racial differences in PD incidence were pointed out, and it is said that PD was less common in China and African countries.^4^

To estimate an accurate annual incidence of PD in Wakayama in 1997 as well as to compare incidence rates between countries, we conducted a mail survey throughout Wakayama in 1998.

## METHODS

A mail survey questionnaire was delivered to each clinic where PD would be potentially diagnosed. The survey was conducted in February 1998 by the Research Committee on Parkinson Disease of Wakayama.^5^ A total of 792 clinics and 87 hospitals were listed as candidates. Physicians in these 879 medical facilities were asked and instructed to register all newly diagnosed cases with PD in 1997 according to the diagnostic criteria proposed by the Japanese Research Committee on Neuro-degenerative Diseases.^6^ (see appendix) When no reply was found, we confirmed representatives of such facilities whether they had new PD cases in 1997 or not. Each questionnaire was verified by one of the authors, a neurologist (SY). After the verification, the cases were checked by initials of their names, sex, date of birth, and address to detect duplicated registries. Finally, one record was registered as a unique case.

The model population proposed by the Japanese government to use for age-adjustment based on the census 1985 was applied as reference population to calculate age-adjusted incidence rates.^7^^,^^8^ According to Kasamatsu et al.,^3^ we divided Wakayama into 3 districts; Kihoku was northern Wakayama with 17 municipalities, Kichu was central Wakayama with 17 municipalities, and Kinan was southern Wakayama with 16 municipalities.

As for background characteristic descriptions, meteorologic information in 1995 was obtained for the 3 districts. Table 1 summarizes the meteorologic differences among 3 districts in Wakayama. Kihoku district showed the coldest climate, while Kichu district had driest air, and Kinan district had the longest sunshine hours.

**Table 1.** Meteorologic background in 3 districts of Wakayama in 1995.

Items	Kihoku	Kichu	Kinan
Location	North	Central	South
Mean temperature (°C)	10.3	16.5	16.7
Total rainfall (mm/year)	1739	1544	2362
Sunshine hours (h/year)	1472	1946	2170

To keep confidentiality of each personal record, we use cases’ initials of names and municipal names of their residences instead of their full names and detailed addresses. Data entry and editing was done only by one of the authors (SM). The ethical approval was given to us independently by the Wakayama Medical Association, the Wakayama Hospital Association, and the Wakayama Medical Research Promotion Foundation.

## RESULTS

Of 879 medical facilities, 873 institutions including 81 hospitals replied to the survey, and the response rate was 99%. A total of 232 cases were reported. Of these, 183 cases were classified as Yahr I to III (YahrI:37; II:76; and III:70). The annual incidence was 16.9 per 100,000 population (95% confidence interval: 14.5-19.3). When age-adjustment with the Japanese model population in 1985 was applied, the figure became 10.5 per 100,000 population (95% CI: 9.3-11.7). Table 2 shows newly diagnosed cases in 1997 by age and sex. Male-to-female ratio was 1:1.4 as a whole, and the dominant age stratum in number was 70 to 79 years old. The highest incidence was 115.6 per 100,000 population in 80 years and over for males, and 88.7 per 100,000 population in 70 to 79 years old for females. Age-adjusted incidence did not show a large sex difference.

**Table 2.** Parkinson disease cases diagnosed in Wakayama 1997.

Age	Cases		Incidence (per 10^5^)		
	
	Male	Female	Male	Female	Total*
Total	75(100%)	108(100%)	10.6**	10.9**	10.5**
[95% CIs]			[8.4-12.8]	[9.0-12.8]	[9.3-11.7]

40-49 years	1( 1)	1( 1)	1.2	1.2	1.2
50-59	6( 8)	9( 8)	8.5	11.9	10.2
60-69	17(23)	33(31)	26.1	45.0	36.1
70-79	35(47)	46(43)	103.4	88.7	94.5
80-	16(21)	19(18)	115.6	64.2	80.6

*: Crude incidence for all cases was 16.9 [95% CI: 14.5-19.3]

**: Age was adjusted using the model population based on the census 1985.

Table 3 summarizes differences of PD incidence by district within Wakayama. The crude rates have an increasing tendency from north to south, similar to Kasamatsu et al. found.^3^ The tendency of age-adjusted rates were attenuated.

**Table 3.** Parkinson disease incidence by district in Wakayama 1997.

Items	Kihoku	Kichu	Kinan
Location	North	Central	South
Incidence [95%CIs] (per 10^5^)	15.9 [12.9-18.9]	18.1 [12.0-24.2]	19.3 [13.4-25.2]
Age-adjusted rate [95% CIs] *	10.8 [ 9.1-12.7]	10.4 [ 8.6-12.2]	9.9 [ 6.9-12.9]

Background features			
municipalities	17	17	16
population in 1995(per 10^5^)	68.5	18.3	21.3
parcentage of 65 years and over	16.3	20.7	21.7

*: Age was adjusted using the model population based on the census 1985.

Table 4 illustrates differences of PD incidence by area, our data together with world figures from previous literatures.^9^^-^^11^ Some early studies included parkinsonism cases, and were suggested two thirds of these figures would be PD cases. This means estimated PD incidences of Rochester were 15.9 and 13.2 per 100,000 population, respectively, and that in Carlisle was 8.1 per 100,000 population. Except an Italian study, the range of PD incidence was not so varied.

**Table 4.** Comparison of Parkinson disease incidence by area.

Area	(Country)	Study period	Incidence (per 10^5^ population)
Wakayama	(Japan)	1997	16.9 [95% CI:14.5-19.3]

Rochester	(USA)	1945-1954	23.8*
Rochester	(USA)	1967-1979	19.7*
Carlisle	(UK)	1951-1961	12.1*
Iceland	(Iceland)	1953-1963	16.0
Sardinia	(Italy)	1961-1971	4.9
Yonago	(Japan)	1975-1978	10.2
Yonago	(Japan)	1989-1992	15.0

*: Parkinsonism cases were included.

## DISCUSSION

In Japan, no survey has been conducted on PD incidence at least by the unit of prefecture.^9^ The largest survey was done twice by Harada’s group in a rural city with around 130,000 population.^10^^,^^11^ Therefore, this is the first incidence study amongst over 1 million Japanese population. From the study results by Harada and Nakashima et al., we could not find age-specific observed populations. This means that we are not able to compare our data directly with theirs through age-adjustment. Interestingly, the estimated annual incidence 16.9 per 100,000 population in Wakayama is almost the same as Harada et al. showed in 1989 to 1992.^10^^,^^11^

We used newly diagnosed PD cases classified as Yahr I to III, not incident cases, in this study. This was because we were uninformed their onset in almost half of the cases. Yanagisawa described a representative natural course of PD based on his clinical experience.^13^ He observed that typical tremor and cogwheel rigidity were the earliest manifestations, and that akinesia and/or festinating gait were not always observed at Yahr I stage. Because Yahr III stage was the first period when all the diagnostic manifestations would be met together, we decided to include Yahr III PD as incident cases.

As we described earlier, patients with PD, Yahr III to V, are financially supported by the rules on intractable diseases in Japan. However, this study suggests that early PD classified as Yahr I and II cases will be as many as those who are now protected by the regulation. These early PD cases will progress into Yahr III or more severe stages, and may need financial supports in the near future. Our findings may give informative suggestions for public health administration.

As for dominant age stratum, Nakashima et al. concluded that newly diagnosed cases between 1988 and 1992 were older than those between 1980 and 1984.^11^^,^^12^ Our findings are compatible with Nakashima’s observations.

Differences of PD incidence within Wakayama attenuated after the age-adjustment. This supports an assumption that differences of crude PD incidence mainly come from differences of district population structure. Ben-Shlomo pointed out that there was little geographic variation in PD incidence and prevalence throughout Europe.^4^ Furthermore, he observed fairly constant incidence in the world except China and West Africa. Our study brings a similar result as Yonago study did by Nakashima et al. These results may support that age structure of the population is more responsible for PD incidence than racial factors. Some neurologic disorders such as multiple sclerosis have been known as climate-related diseases.^14^ Although meteorologic differences in three districts in Wakayama were striking, PD incidence was fairly constant just after the age-adjustment. This again supports that the population structure is more responsible for the differences of PD incidence in Wakayama. As for international comparison, age-specific incidence was not available. We conclude that we could not compare PD incidence in different countries directly by using published data at the moment.

There are some limitations in our study. First, the study method was not the same as other previous studies. We understand that community door to door studies are more reliable, but this method would not always be available when we survey over 1 million population. Second, there is a possibility that we underestimated the number of PD cases who were diagnosed at medical facilities outside Wakayama. As far as PD patients who receive financial aid for Yahr III to V in Wakayama are referred, we know that 93% of PD cases in 1997 were diagnosed inside Wakayama.^15^ Thanks to the members of the Wakayama Medical Association, we achieved a high response rate over 99%. However, 6 hospitals did not reply even after being reconfirmed 3 times. Of these hospitals, 3 were in Kihoku district, 1 was in Kichu, and 2 were in Kinan. The 3 un-replied hospitals in Kihoku did not have a department of neurology. The unanswered Kichu hospital was specialised in cardiovascular and gastroenterologic diseases. The 2 hospitals in Kinan were the main general hospital with a department of neurology, and its branch with a psychiatry division. This means that cases reported from Kinan district would be smaller in number than real cases, while those from Kihoku and Kichu would be reported more properly. The effect of under-report in Kinan district is not clear, but is thought to be at least not to dilute differences of incidence by district. Therefore we believe that our study provided fairly accurate estimation.

Our method was simple and not expensive, but we think that the results were worth using for the public health administration.

## CONCLUSION

The annual incidence of PD in Wakayama, Japan was estimated as 16.9 per 100,000 population (95% CI: 14.5-19.3). The figure became 10.5 per 100,000 population (95% CI: 9.3-11.7) after the age-adjustment. The age-adjusted incidence did not show a large difference between the sexes. The increasing tendency in PD incidence from north to south within Wakayama was disappeared when age-adjustment was applied.

## APPENDIX

Diagnostic criteria for Parkinson disease proposed by the Japanese Research Committee on Neuro-degenerative Diseases.

1. Unclear onset with slowly progressive course after 20 years old.
2. At least one manifestation of a) - c) is present.
  a) Typical 4-6 Hz tremor
  b) Cogwheel rigidity with akinesia and/or festinating gait
  c) If a) and b) are absent, three of postural tremor, lead-pipe rigidity, akinesia, festinating gait, and retropulsion are present.
3. L-DOPA is effective for improving physical activity of the patient.
4. No medication of parkinsonism-induced drugs.
5. Exclude parkinsonism such as cerebro-vascular, post-encephalitic, manganese poisoning, post-traumatic, normal pressure hydrocephalus, striato-nigral degeneration, Shy-Drager syndrome, progressive supranuclear palsy, progressive pallidum atrophy, Creutzfeldt-Jakob disease.
